# Substrate and Target Sequence Length Influence RecTE_Psy_ Recombineering Efficiency in *Pseudomonas syringae*

**DOI:** 10.1371/journal.pone.0050617

**Published:** 2012-11-30

**Authors:** Zhongmeng Bao, Sam Cartinhour, Bryan Swingle

**Affiliations:** 1 Department of Plant Pathology and Plant-Microbe Biology, Cornell University, Ithaca, New York, United States of America; 2 United States Department of Agriculture-Agricultural Research Service, Ithaca, New York, United States of America; University of the West of England, United Kingdom

## Abstract

We are developing a new recombineering system to assist experimental manipulation of the *Pseudomonas syringae* genome. *P. syringae* is a globally dispersed plant pathogen and an important model species used to study the molecular biology of bacteria-plant interactions. We previously identified orthologs of the lambda Red *bet/exo* and Rac *recET* genes in *P. syringae* and confirmed that they function in recombineering using ssDNA and dsDNA substrates. Here we investigate the properties of dsDNA substrates more closely to determine how they influence recombineering efficiency. We find that the length of flanking homologies and length of the sequences being inserted or deleted have a large effect on RecTE_Psy_ mediated recombination efficiency. These results provide information about the design elements that should be considered when using recombineering.

## Introduction

*Pseudomonas syringae* is a model species used to study the molecular biology of bacteria-plant interactions and mechanisms that bacteria use to grow parasitically and cause disease. *P. syringae* is a hemibiotrophic pathogen that can grow epiphytically on plants and in other environments, then switch to a parasitic lifestyle when the correct environmental conditions are encountered [1], [2]. The *P. syringae* species comprises over 50 pathovars based on the range of plants that individual strains can infect [3]. For example, *P. syringae* pv. *tomato* DC3000 is able to cause disease in tomato (*Solanum lycopersicum*) and *Arabidopsis thaliana*. The genomes of *P. syringae* pv. *tomato* DC3000 [4] and both hosts have been fully sequenced [5], [6], facilitating detailed analysis of their interactions.

Phage-encoded recombination enzymes catalyze a form of homologous recombination that can be used for recombineering, a powerful approach for *in vivo* site-specific modification of bacterial genomes [7]. These reactions are gene conversion events that result in alteration of genomic loci to match the sequence of a substrate DNA introduced into the cell by electroporation. When the substrate DNA is double stranded, two phage-encoded functions cooperate to carry out the reaction. The dsDNA is first made single stranded by a 5′->3′ exonuclease [8], [9], [10], [11]. The newly formed ssDNA is then bound by a ssDNA annealing protein that protects the ssDNA from degradation and promotes annealing at the homologous target location [10], [11], [12], [13]. The phage-encoded proteins appear to have evolved to take advantage of a feature of the replisome that allows ssDNA to direct changes at homologous targets in the genome without RecA [14], [15], [16], [17].

In a previous study, we identified *recTE*_Psy_ genes encoded by *P. syringae* pv. *syringae* B728a and demonstrated that the *recT*_Psy_ gene product can facilitate ssDNA recombination and that RecTE_Psy_ are necessary for dsDNA recombineering in *P. syringae* pv. *tomato* DC3000 [18]. Based on protein sequence features and functional analysis, it is likely that *recTE_Psy_* encode *P. syringae* orthologs of the well-studied phage lambda Red proteins Beta/Exo [18], [19], [20] and the RecT/RecE proteins of the *Escherichia coli* Rac prophage [18], [21], [22]. In this study we compared a variety of dsDNA substrates and their recombination frequencies to determine how substrate design affects recombineering efficiency in the *P. syringae* DC3000 RecTE_Psy_ system.

## Materials and Methods

### Bacterial Strains and Growth Conditions

*Pseudomonas syringae* strains were grown at 30°C in Kings B (KB) medium [23] or on KB medium solidified with 1.5% (wt/vol) agar. Gentamycin and kanamycin were used at 10 µg/ml and 50 µg/ml respectively. *E. coli* DH5α was used as the host for subcloning and other plasmid manipulations. *E. coli* was grown at 37°C in LB medium or LB medium solidified with 1.5% (wt/vol) agar. For a complete list of strains and plasmids used in this work see Supporting Information Table S1.

### Recombineering Substrates

Recombineering substrates were produced by PCR. The sequences of all oligos used are shown in Supporting Information Table S2. Oligos were purchased from Integrated DNA Technologies (IDT), Inc., Coralville, IA. PCR products were generated using ExTaq (Takara Bio, Inc, Japan). Fragment size and integrity for all PCR generated substrates were confirmed by agarose gel electrophoresis and products purified and concentrated by ethanol precipitation. Recombineering substrates were generated using pK18mobsacB [24], ΔPSPTO1203::*neo* or pZB111 as PCR templates. ΔPSPTO1203::*neo* was constructed by RecTE_Psy_ recombineering using 1 µg of PCR product (oSWC2648/2649 with pK18mobsacB as template) that had been digested with lambda exonuclease (NEB, Ipswich, MA) *in vitro*. Note, lambda Exo was not used to pre-digest any other substrates described in this manuscript. pZB111 was constructed by ligation of Bsu36I digested pACYC184 to the Bsu36I digested PCR product of oSWC4347/4349 using pK18mobsacB as template. All constructs and strains used as templates to produce recombineering substrates were confirmed by restriction digestion and/or sequencing.

### Recombineering P. Syringae pv. Tomato DC3000

Electrocompetent *P. syringae* pv. *tomato* DC3000 transformed with pUCP24/recTE or the empty vector control pUCP24/61 were prepared using the method described by Swingle et al., 2010 [18]. The pUCP24/recTE plasmid contains the *recTE*_Psy_ genes cloned downstream of the *P_nptII_* promoter, which provides constitutive expression of the recombineering functions [18]. To prepare electrocomptetent cells, *P. syringae* cultures were grown to an OD_600_ of 0.6–0.8 in KB medium [23], cells were harvested by centrifugation at room temperature, washed twice with equal volume of room temperature 300 mM sucrose and resuspended in 1/60 volume 300 mM sucrose. In each experiment the indicated amount of PCR product was added to 100 µl of electrocompetent cells and transformed by electroporation at 2.5 kV, 25 µF, 200 Ω in a 0.2 cm cuvette using a Gene-pulser (Bio-Rad Laboratories, Hercules CA). KB medium (4.0 ml) was then added and cells were incubated with shaking at 30°C overnight. To determine the recombination frequency, dilutions of transformation outgrowth cultures were spread on selective (50 µg/ml kanamycin) or non-selective KB-agar plates. To facilitate comparisons between experiments, results are expressed as the number of kanamycin resistant transformants per 10^8^ viable cells. Recombination frequencies are the average of at least three independent experiments and error bars indicate standard deviation. In experiments where recombineering efficiency was assessed, targeting fidelity was determined in kanamycin resistant cells using PCR with primers that flank the targeted locus. Primers were designed to yield different length products depending on whether mutant or wild-type alleles were present. Product lengths were determined using agarose gel electrophoresis.

## Results

A simple and widely used approach for constructing mutant strains involves introducing an antibiotic resistance gene at a designated locus. Recombineering offers an efficient strategy to produce this type of mutation. In the experiments described here, cells expressing RecTE_Psy_ were transformed with PCR-generated linear DNA molecules containing an antibiotic resistance gene, flanked by genomic homologies to direct recombination to a specific locus. Recombinants were selected using the corresponding antibiotic.

### Substrate Concentration

**Figure 1:**
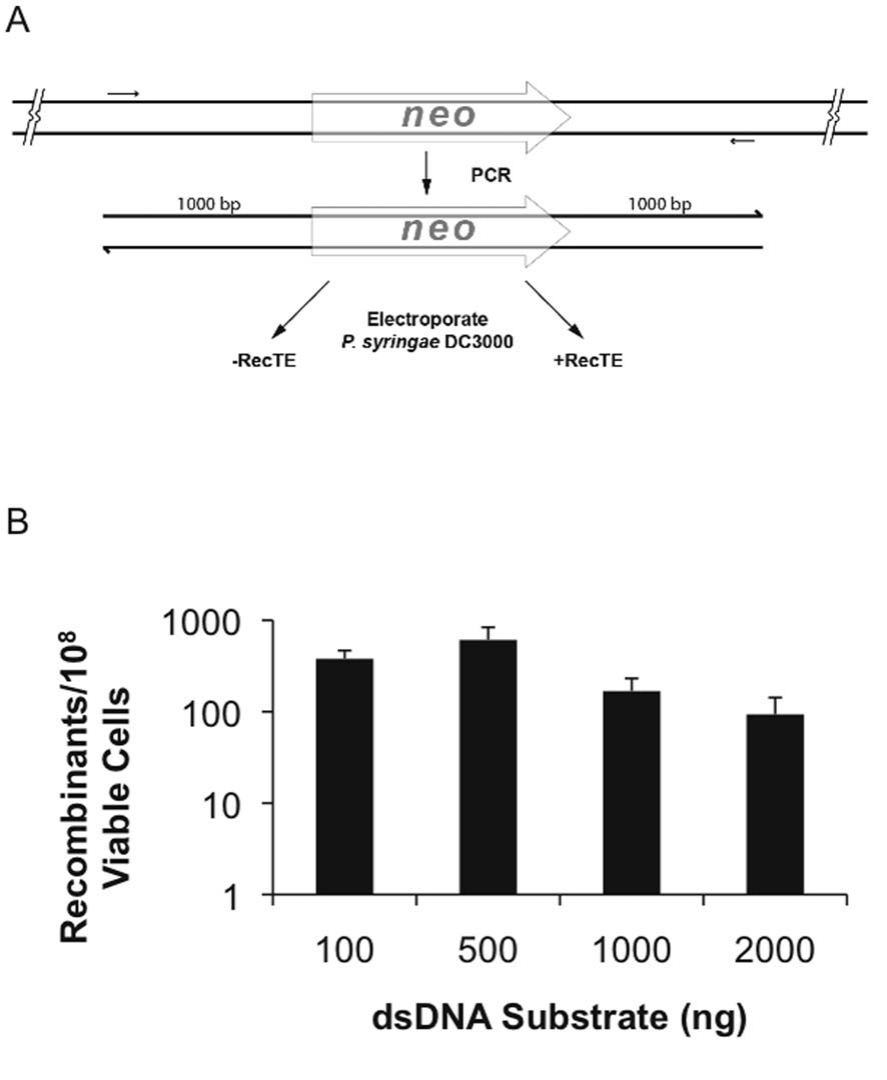
The amount of dsDNA substrate influences RecTE_Psy_ recombination frequency. The amount of PCR product added for electroporations was varied in order to determine the effect of substrate concentration on recombination. (A) Recombineering substrates were generated using PCR. The PCR product had 1 kb flanks identical to target location (the PSPTO_1203 locus) at each end of the *neo* gene encoding kanamycin resistance. This substrate deletes 500 bp of the 543 bp PSPTO_1203 gene and inserts the 1.3 kb *neo* gene in its place. (B) Maximum recombination frequency was observed when 500 ng of PCR product was added to the electroporations with RecTE_Psy_ present. The results are the average of four independent replicates, error bars indicate standard deviation. No kanamycin resistant colonies were observed in the absence of the RecTE_Psy_ expression vector (data not shown).

The amount of dsDNA added to the transformation mix was varied to determine the effect of substrate concentration on the frequency of RecTE_Psy_ mediated recombination. The dsDNA substrate contained the *neo* gene encoding kanamycin resistance [24] flanked by 1 kb regions with homology to sequences adjacent to the PSPTO_1203 locus (Fig. 1A). *P. syringae* DC3000 strains [18] containing either the RecTE_Psy_ expression vector (pUCP24/recTE) or the empty vector control (pUC24/61) were transformed with varying amounts of PCR product and the number of kanamycin resistant recombinants was determined (Fig. 1B). No kanamycin resistant colonies were obtained in cells containing the empty vector control, demonstrating a strict requirement for RecTE_Psy_ for recombination using this type of substrate. In cells with RecTE_Psy_ we observed two levels of recombination. Higher recombination frequencies were observed in transformations using 100 ng or 500 ng of substrate, while the frequency decreased with 1.0 µg and 2.0 µg of substrate. For example, recombination frequency with 500 ng of substrate was 6.5 fold higher than with 2 µg of substrate. Substrate concentration similarly affects Red mediated recombination [20].

These results indicate that RecTE_Psy_-mediated recombination of dsDNA is more efficient when relatively low amounts of substrate are used and that increasing the amount of substrate reduces recombination frequency. This contrasts with the RecT_Psy_-mediated recombination of ssDNA oligonucleotides, where maximum recombination frequency is obtained with higher levels of substrate [18]. The association between maximal recombination frequencies and lower dsDNA concentrations may be a consequence of exonuclease (RecE)-assisted loading of ssDNA annealing protein (RecT) onto newly exposed ssDNA [22], [25]. In contrast, when single-stranded DNA oligos are introduced into the cell by electroporation, they are not expected to benefit from RecE-assisted loading, and therefore require more substrate to compensate for less RecT protection.

### Flanking Homology Length

**Figure 2:**
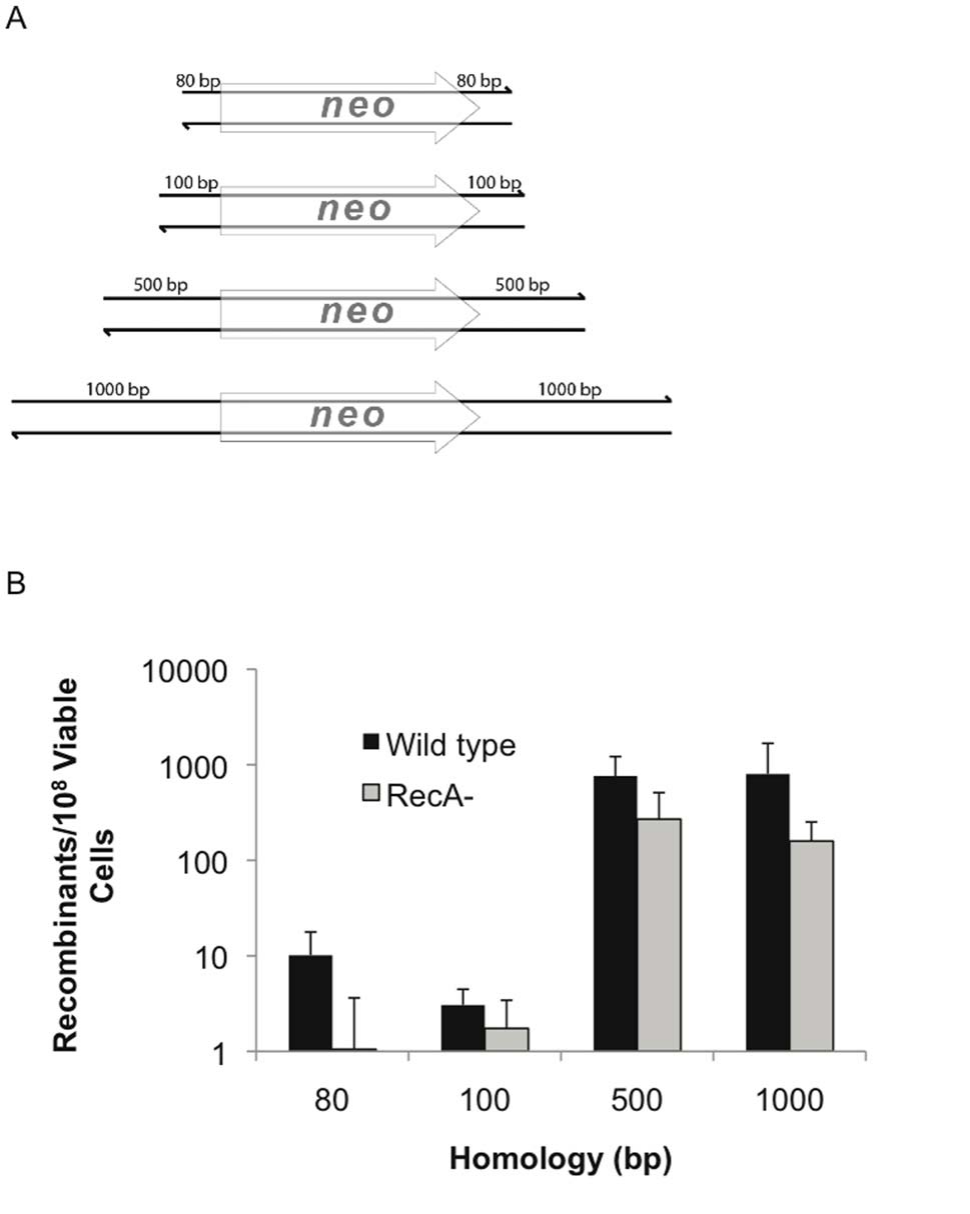
RecTE_Psy_-mediated recombination frequency is sensitive to flank length. (A) Recombination substrates were generated by PCR using primers that flank the ΔPSPTO_1203::*neo* allele to produce substrates with homologies of indicated lengths. For example, each of the substrates is composed of the *neo* gene flanked by the indicated amount of genomic sequence. The substrate was then gel purified and 0.46 pmol of each substrate was used to electroporate cells containing the RecTE_Psy_ expression vector or empty vector (0.46 pmol corresponds to 200 ng of the 80 bp flanks substrate). (B) Recombination frequencies with these substrates in wild-type and *recA* ^−^ *P. syringae* pv. *tomato* DC3000 with pUCP24/recTE are shown. The results are the average of at least three independent replicates, error bars indicate standard deviation. No kanamycin resistant colonies were observed in control transformations of cells containing the pUCP24/61 empty vector (no RecTE_Psy_). PCR was used to confirm that for each length tested the kanamycin resistance gene had integrated into the correct location.

The effect of homology length on recombination frequency was assessed by transforming *P. syringae* DC3000 cells containing the RecTE_Psy_ expression plasmid (pUCP24/recTE) or the empty vector control (pUCP24/61) with PCR products of various lengths while keeping substrate molarity constant. Recombineering substrates were generated using the ΔPSPTO_1203::*neo* locus as a PCR template, with primers positioned to generate a symmetrical product with two flanks of equal length on each side of the *neo* gene. Flank lengths ranged in size from 80 bp to 1 kb (Fig. 2A). A dramatic (250-fold) increase in efficiency was observed as flanks were extended from 100 to 500 bp, but no further boost in efficiency was seen with 1 kb flanks (Fig. 2B).

The strong correlation with flank length and recombination frequency was somewhat surprising given that 100 bp homology flanks are sufficient to give maximal recombination efficiency with the *E. coli* RecET recombineering system [22] and 40 bp flanks enable near-maximal levels of Red-mediated recombination [20]. Finding that longer substrates recombined more efficiently suggested the possibility that RecA might be assisting these reactions. RecA requires homologies longer than 200 bp to function efficiently [26]. However, eliminating RecA did not preferentially reduce the recombination frequency for long substrate lengths, supporting the hypothesis that RecA is not responsible for increased recombination of the longer homology substrates. The general reduction in recombination frequency observed in the absence of RecA is thought to be due to a decrease in viability of this mutant as has been reported for lambda Red [20]. These results suggest that these recombination events are RecA independent, as is typical for this type of recombineering [20].

To explain the flank length observations we speculated that cellular nucleases might limit recombination by degrading incoming DNA and compromising the homology flanks of shorter substrates [16], [27]. The amount of recombineering substrate that is degraded can be minimized by co-transforming a large excess of non-specific carrier DNA along with the recombineering substrate, presumably because the carrier overwhelms the capacity of the nucleases [15], [27]. To test this possibility, we transformed cells with the 100 bp flank substrate and included 1.0 µg or 2.0 µg of non-homologous double stranded PCR product (PSPTO_3957) or ssDNA oligonucleotides (oSWC1447) as carrier. The addition of carrier did not increase the recombination frequency (data not shown), suggesting that degradation is not responsible for the different recombination frequencies of long and short substrates.

**Figure 3:**
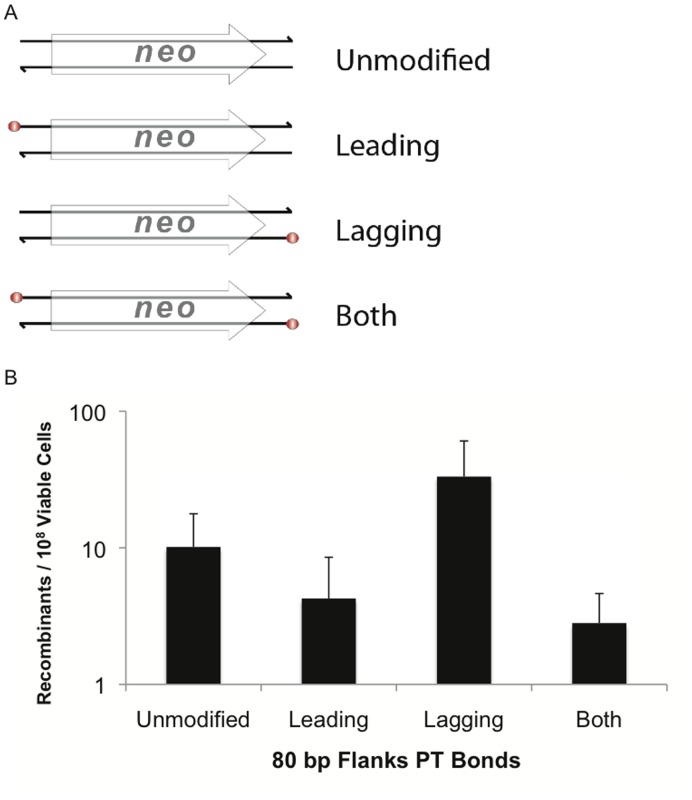
Phosphorothioate bonds on the matching lagging strand provide a modest increase in recombineering. (A) Locations of the 5′ phosphorothioate bonds at the four terminal nucleotides are indicated as red spheres. Leading, Lagging and Both indicate the location of the 5′ phosphorothioate bonds on the respective strands of the substrates relative to the target location. (B) Recombination frequency for 80 nt flank protected substrates. 200 ng of each substrate was used in each electroporation. The results are the average of at least four independent replicates, error bars indicate standard deviation.

Modifying the 5′ ends with phosphorothioate (PT) bonds protects substrate DNA from exonucleolytic degradation and can increase recombination frequency [22], [28]. To examine the role of substrate degradation more closely, we generated 80 bp flank substrates containing four 5′ PT bonds and compared their recombination efficiency (Fig. 3). A modest (3-fold) increase was observed with substrates containing PT bonds positioned to protect the strand matching the lagging strand. However, the recombination frequency was 2.4- or 3.6-fold lower with substrates containing PT bonds that protect either the leading strand or both strands, respectively. These results suggest that the benefit of PT modification comes from biasing degradation of the leading strand by RecE_Psy_, rather than protecting the substrate from general degradation. This would leave a greater concentration of lagging strand substrate available to direct recombination more efficiently. In contrast, a similar experiment using lambda Red showed that modifying only the lagging targeting strand or both strands increased recombination frequency [10]. The disparity may reflect differences in the efficiency of the nucleases in these two species or differences in the way that the recombinase enzymes function.

### Substrate and Target Geometry Influence Recombination Frequency

**Figure 4:**
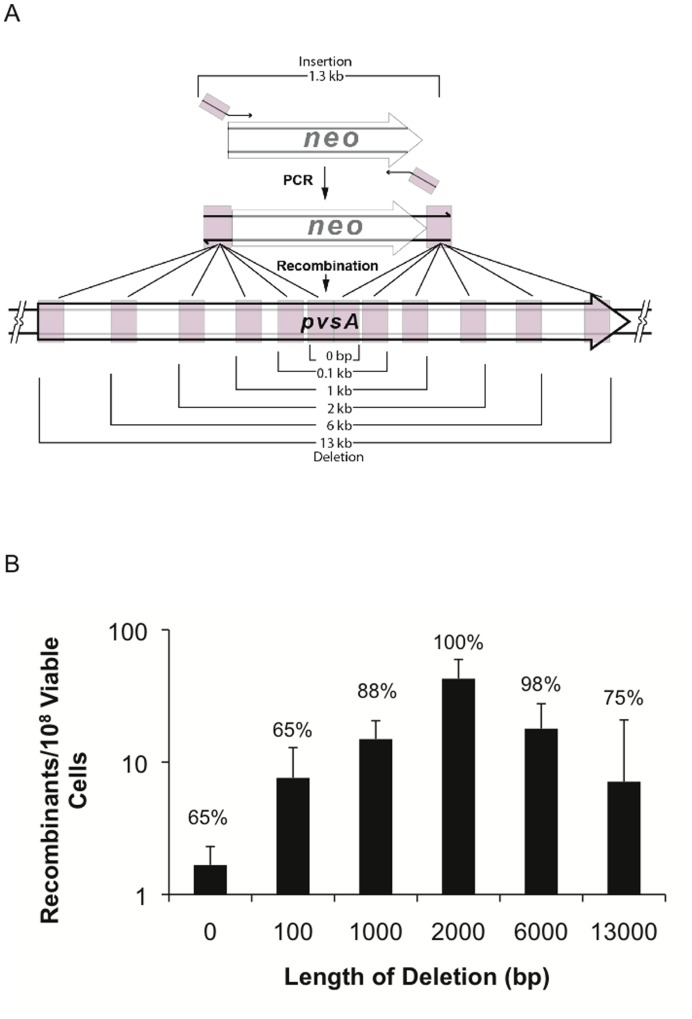
Deletion size affects RecTE_Psy_-mediated recombination efficiency. (A) Recombination substrates were generated using PCR primers to amplify the *neo* gene with 80 nt homologies to the *pvsA* gene at the ends of the PCR product. The size of the deletion is determined by the distance between the *pvsA* flanking sequences as they exist on the *P. syringae* DC3000 genome. Homologous sequences are shown in pink. 500 ng of each substrate was used in electroporations. (B) Recombineering efficiency was determined for each substrate. Between 20 to 60 kanamycin resistant clones from each deletion length were analyzed using PCR to determine whether the *neo* gene had integrated and produced a deletion in the correct location. The percentage of kanamycin resistant clones with the correct deletion is indicated above each bar. The results are the average of four independent replicates, error bars indicate standard deviation.

RecTE_Psy_ mediated recombineering makes it possible to construct different size deletions depending on the position of flanking homologies in the genome. The relationship between the amount of sequence being deleted and recombineering efficiency was analyzed by constructing a series of different length deletions in the 13 kb *pvsA* gene. This gene encodes a nonribosomal peptide synthetase for the pyoverdine chromophore. The *pvsA* gene was chosen because it can accept a wide range of deletion sizes without complicating the comparisons by altering the sequence of neighboring genes. The deletions were directed using 1.3 kb PCR products that contained the *neo* gene and 80 bp flanks homologous to the *pvsA* gene (Fig. 4A). Short flanking sequences are advantageous because they can be introduced using a single PCR.

To evaluate the recombineering efficiency, we measured the recombination frequency for each substrate and determined the proportion of kanamycin resistant clones that received the recombineering modification in the correct target location. A wide range of recombination efficiencies was observed depending on the size of the deletion being directed (Fig. 4B). The 2 kb deletion was the most efficient, in terms of facilitating the highest recombination frequency (42.7 recombinants per 10^8^ viable cells) and maximum targeting fidelity; 100% (n = 50) of the deletions occurred precisely as directed by the recombineering substrate in each of the five replicates of this experiment. Interestingly, deletion length and recombineering efficiency do not appear to be linearly correlated. The 6 kb deletion was nearly as efficient as a 2 kb deletion (2.4-fold difference), suggesting that long deletions are tolerated and we were able to reliably produce deletions up to 13 kb. In contrast recombineering efficiency decreased rapidly for deletions shorter than 2 kb.

**Figure 5:**
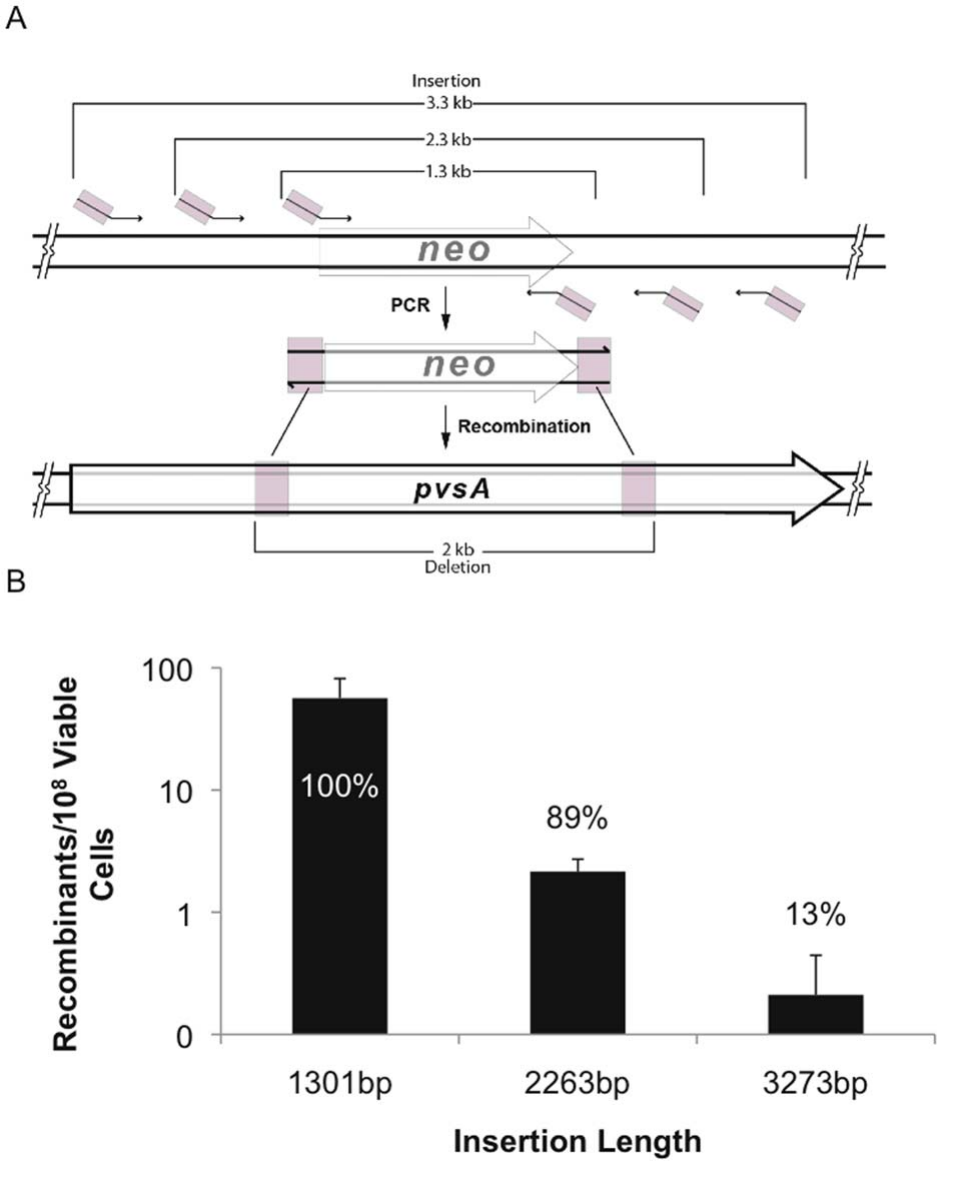
Insertion length influences recombination efficiency. (A) The length of the region between the homologous flanking sequences was varied to evaluate whether its length affects recombination frequency. Substrates were designed to generate a 2 kb chromosomal deletion upon insertion of the length indicated. Homologous sequences are shown in pink. (B) Recombineering efficiency with substrates that insert different amounts of sequence. PCR was used to analyze the *pvsA* alleles of 8–18 kanamycin resistant clones for each deletion length to determine whether the *neo* gene had integrated and produced the deletion in the correct location. The percentage of kanamycin resistant clones with the correct deletion is indicated above each bar. The results are the average of three independent replicates, error bars indicate standard deviation.

We also examined whether length effects were apparent when recombineering was used to generate different length insertions. The recombineering substrates for this experiment were generated by PCR of pZB111, which contains the *neo* gene cloned in pACYC184 (see Materials and Methods), using primers that include 80 nt of homology to the *pvsA* locus. The products direct a 2 kb deletion of the *pvsA* gene. The primers were chosen so that the *neo* gene would be amplified along with varying amounts of vector sequence (Fig. 5A). The template does not contain any substantial homologies to the *P. syringae* DC3000 genome or the pUCP24/*recTE* expression vector (confirmed by Blast analysis, data not shown). A dramatic reduction in recombination frequency and targeting fidelity was observed as insert length was increased above 1.3 kb (Fig. 5B). The recombination frequency of the 1.3 kb insert was 26- and 268-fold higher than the 2.3 kb and 3.3 kb substrates, respectively. Lambda Red recombineering displays a similar sensitivity to insertion length, possibly due to Exo processivity [11].

**Figure 6:**
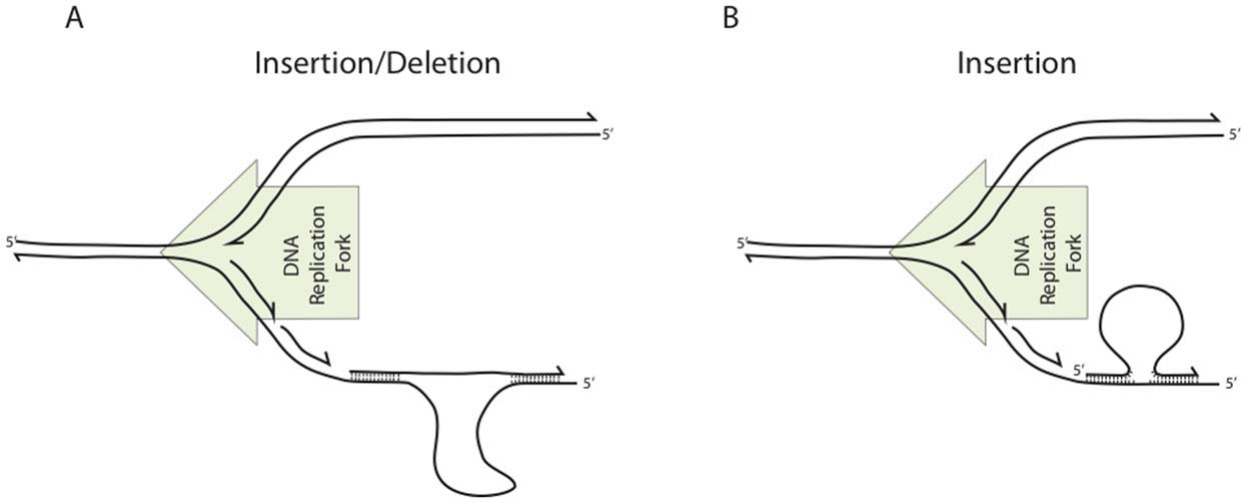
Models of target-substrate structures. (A) When the segment of the target molecule being deleted is larger than the segment being inserted, the target must bend to permit target-substrate annealing. For short insertions, this reaction has higher recombination efficiency, suggesting that the genome more easily adopts a favorable structure. (B) When a recombineering substrate directs an insertion without concomitant deletion, the substrate must bend to achieve target-substrate annealing. The flexibility of the substrate is likely to influence the stability of recombineering heteroduplex intermediate. The green arrow indicates the direction of DNA replication fork movement.

The results of the deletion and insertion length experiments suggest that the relationship between the substrate and the genomic sequence being altered strongly affects the efficiency of the recombineering reaction. When the length of the sequence cargo on the recombineering substrate is shorter than the region of the genome being deleted, the reaction is favored. Conversely, when the cargo is longer, the reaction is impaired (Fig. 6). One possible explanation is that bending or looping of the genome is favored over bending of the recombineering substrate.

## Discussion

The work described here was motivated by a desire to learn which details of substrate design are most critical to RecTE_Psy_ recombineering success using PCR products containing an antibiotic resistance gene. We were able to efficiently recover mutants containing a wide variety of insertions and or deletions at specific loci and observed that the recombination frequency and target specificity decrease as insertion and deletion size limits are approached. Size constraints have not been a factor for most mutants that we have constructed for routine work. However, the data show that mis-targeting occurs in some cases and that PCR screening is required to identify correct constructs. It is possible that some aspect of target sequence or context may influence the effect of insertion and deletion length on recombination efficiency; however, more work will be required to understand these dynamics.

These experiments also offer insights regarding reaction intermediates when recombineering substrates anneal to the genome. (Fig. 6) First, we found that the strand bias, which appears to apply universally to all recombineering systems [29], [30], [31], [32], [33] as well as phage recombinase independent oligo recombination [15], also applies to RecTE_Psy_ (Fig. 3). This is consistent with our hypothesis that the phage encoded recombinases have evolved to take advantage of an inherent attribute of the replisome to catalyze recombination [15], possibly the larger region of ssDNA exposed on the lagging template strand [30], [31].

Recent evidence suggests that a dsDNA recombineering substrate is converted to a fully single stranded intermediate to facilitate annealing to the genomic target at the replication fork [10], [11]. Our results demonstrate that the target homologies can be far apart in the genome sequence and that a large region can be deleted (Fig. 4). For large deletions (Fig. 6A), the two genomic regions participating in the reaction may not be available in single-stranded form at the same time because of their position relative to the replication fork. In cases where the single stranded intermediate matches the lagging strand, the 3′ end of the substrate first anneals to the homologous sequence and remains in this partially annealed state until the region matching the 5′ end of the substrate is exposed. Additionally, when inserting an antibiotic resistance gene, a region of heteroduplex sequence is predicted to form as the substrate anneals to the target [11], [27]. The heteroduplex must be sustained through a second round of replication in order for the two strands to segregate, giving rise to one recombinant and one parental cell. Accordingly, the stability of the heteroduplex may impose constraints on some insertions and deletions.

The heteroduplex must take different conformations depending on the relationship between the length of the substrate and target. When the region of the chromosome being deleted is longer than the sequence cargo carried on the recombineering substrate, the chromosome must form a loop or a bend to allow the substrate flanks to anneal to the genome (Fig. 6A). Our results show that long deletions are much more favored than long insertions, suggesting that chromosome bending is preferred to substrate bending. Conversely, when the region of the genome being deleted is shorter than the recombineering substrate (for example, in the case of large insertions), we observe a decrease in recombination frequency and an increase in non-target site integration. This may be due to inherent rigidity of short DNA molecules or the rigidity of the RecT_Psy_-DNA filament complex (Fig. 6B).

We do not yet understand the basis of mis-targeting and have not determined the location of the corresponding *neo* gene integration sites, but interpret it as an indication that RecTE_Psy_ enabled recombination is compromised with some substrates. Integration at non-target sites is observed with Red-mediated recombineering as well. Mosberg et al. proposed that mis-targeting might be due to microhomologies between the recombineering substrate and non-target sites in the genome, but the factors that influence mis-targeting have not been investigated [10].

## Supporting Information

Table S1 **Strains and plasmids used.**
(DOCX)

Table S2 **Oligonucleotides used.**
(DOCX)
