# Supplementary material for: Substrate and Target Sequence Length Influence RecTEPsy Recombineering Efficiency in Pseudomonas syringae
Source: PLoS One. 2012 Nov 30;7(11):e50617. doi: 10.1371/journal.pone.0050617 (PMC3511549; doi:10.1371/journal.pone.0050617)
Supplement: Table S1 — Strains and plasmids used. (DOCX) [file pone.0050617.s001.docx]

**Supporting Information**

| Strain/Plasmid | Description | Reference |
| --- | --- | --- |
| *P. syringae* pv. *tomato* DC3000 | Wild-type | [[1](#_ENREF_1)] |
| *P. syringae* pv. *tomato* DC3000  ΔPSPTO1203::*neo* | ΔPSPTO1203::*neo* | This work |
| *E. coli* DH5α | F– Φ80*lac*ZΔM15 Δ(*lac*ZYA-*arg*F) U169 *rec*A1 *end*A1 *hsd*R17 (rK–, mK+) *pho*A *sup*E44 λ– *thi*-1 *gyr*A96 *rel*A1 | [[2](#_ENREF_2)] |
| pUCP24/recTE | P*_nptII_*::*recTE*_Psy_ | [[3](#_ENREF_3)] |
| pUCP24/61 | P*_nptII_* promoter cloned upstream of a multiple-cloning site; used as the empty vector control | [[3](#_ENREF_3)] |
| pZB111 | pACYC184 derivative carrying neo from pK18mobsacB | This work |
| pACYC184 | Cloning vector | [[4](#_ENREF_4)] |
| pK18mobsacB | Source of *neo* gene | [[5](#_ENREF_5)] |

Table S1. Strains and plasmids used.

**References**

1. Buell CR, Joardar V, Lindeberg M, Selengut J, Paulsen IT, Gwinn ML, Dodson RJ, Deboy RT, Durkin AS, Kolonay JF, Madupu R, Daugherty S, Brinkac L, Beanan MJ, Haft DH, Nelson WC, Davidsen T, Zafar N, Zhou LW, Liu J, Yuan QP, Khouri H, Fedorova N, Tran B, Russell D, Berry K, Utterback T, Van Aken SE, Feldblyum TV, D'Ascenzo M, Deng WL, Ramos AR, Alfano JR, Cartinhour S, Chatterjee AK, Delaney TP, Lazarowitz SG, Martin GB, Schneider DJ, Tang XY, Bender CL, White O, Fraser CM, Collmer A (2003) The complete genome sequence of the Arabidopsis and tomato pathogen *Pseudomonas syringae* pv. *tomato* DC3000. Proceedings of the National Academy of Sciences of the United States of America 100 (18):10181-10186

2. Hanahan D (1983) Studies on transformation of *Escherichia coli* with plasmids. J Mol Biol 166 (4):557-580

3. Swingle B, Bao Z, Markel E, Chambers A, Cartinhour S (2010) Recombineering Using RecTE from *Pseudomonas syringae*. Appl Environ Microbiol 76 (15):4960-4968

4. Chang AC, Cohen SN (1978) Construction and characterization of amplifiable multicopy DNA cloning vehicles derived from the P15A cryptic miniplasmid. J Bacteriol 134 (3):1141-1156

5. Schafer A, Tauch A, Jager W, Kalinowski J, Thierbach G, Puhler A (1994) Small mobilizable multi-purpose cloning vectors derived from the *Escherichia coli* plasmids pK18 and pK19: selection of defined deletions in the chromosome of Corynebacterium glutamicum. Gene 145 (1):69-73
