# Supplementary material for: Substrate and Target Sequence Length Influence RecTEPsy Recombineering Efficiency in Pseudomonas syringae
Source: PLoS One. 2012 Nov 30;7(11):e50617. doi: 10.1371/journal.pone.0050617 (PMC3511549; doi:10.1371/journal.pone.0050617)
Supplement: Table S2 — Oligonucleotides used. (DOCX) [file pone.0050617.s002.docx]

| ID | Description | Sequence |
| --- | --- | --- |
| oSWC2648 | Forward PCR primer, 81 nt PSPTO_1203, 0.5 kb deletion, *neo* insertion | AGATTTTGATGTCATCTTTCGCGTTAATGATAATTTTTCGTGTTTGAGAAGCTTTGACGTATGCGCGACTTTTCCGCCGTTTGCCGCAAGCACTCAGGGCGCAAGG |
| oSWC2649 | Reverse PCR primer, 81 nt PSPTO_1203, 0.5 kb deletion, *neo* insertion | AGGCCAGCCAGGAAGCGGCCTCGGTGATCATTCGTTCGGAGGGTTCACGCTGGTCGCTCATGCAAAATGGCCCGAACCATGTCAGAAGAACTCGTCAAGAAGGCG |
| oSWC2712 | Forward PCR primer ΔPSPTO_1203:*neo*, 1000 bp flank | AGTGGCCGTGAACCGGCACGTAG |
| oSWC2713 | Reverse PCR primer ΔPSPTO_1203:neo, 998 bp flank | TTTATCAGCGCTGTGTCATCG |
| oSWC4306 | Forward PCR primer ΔPSPTO_1203:*:neo*,  80 bp genomic flank | GATTTTGATGTCATCTTTCGCG |
| oSWC4306* | Forward PCR primer ΔPSPTO_1203:*:neo*, 80 bp genomic flank, 4 5’ phosphorothioates | G*A*T*T*TTGATGTCATCTTTCGCG |
| oSWC4307 | Reverse PCR primer ΔPSPTO_1203:*:neo*, 81 bp genomic flank | AGGCCAGCCAGGAAGCGGCCTCGGTGATCATTC |
| oSWC4307* | Reverse PCR primer ΔPSPTO_1203:*:neo*, 81 bp genomic flank, 4 5’ phosphorothioates | A*G*G*C*CAGCCAGGAAGCGGCCTCGGTGATCATTC |
| oSWC2708 | Forward PCR primer ΔPSPTO_1203::*neo*, 100 bp genomic flank | TCCTGTGCTTGCTGCTGACAG |
| oSWC2709 | Reverse PCR primer ΔPSPTO_1203:*:neo*, 100 bp genomic flank | ACCGGCTCATCCTGAAGCAAGG |
| oSWC4304 | Forward PCR primer ΔPSPTO_1203:*:neo*, 500 bp genomic flank | AAATGTCGTCAGATGGCGGCGG |
| oSWC2711 | Reverse PCR primer ΔPSPTO_1203:*:neo*, 499 bp genomic flank | TCGACCCACAATTCGCCTTG |
| oSWC4324 | Forward PCR primer, 80 nt *pvsA*, 0 bp deletion, *neo* insertion | TGCCGCCTCTGCTGCAACTGTTCATCGACGAACCGCTGACGCAGCAGTGCAGTAGCCTGCGTCGCCTGTTCTCCGGCGGCGTGCCGCAAGCACTCAGGGCGCAAGG |
| oSWC4325 | Reverse PCR primer, 80 nt *pvsA*, 0 bp deletion, *neo* insertion | TCGGGCCATAACGGTTGTGCAACTGCACGTTCGGCAGTTGCTCCAGCACCCGGTTGCGCAGTTCACTCGGCAGCGCTTTCAGAAGAACTCGTCAAGAAGGCG |
| oSWC4326 | Forward PCR primer, 80 nt *pvsA*, 100 bp deletion, *neo* insertion | ACCGAATCGCGCAACTGGTCAACGAATATGGCGTGACCACGCTGCATTTCGTGCCGCCTCTGCTGCAACTGTTCATCGACGATGCCGCAAGCACTCAGGGCGCAAGG |
| oSWC4327 | Reverse PCR primer, 80 nt *pvsA*, 100 bp deletion, *neo* insertion | AGGCCATCGGCACGCAGGCACTGCCAATGAGTGACGTTGATCGCGGTTTCGGTCGGGCCATAACGGTTGTGCAACTGCACTCAGAAGAACTCGTCAAGAAGGCG |
| oSWC4328 | Forward PCR primer, 80 nt *pvsA*, 1 kb deletion, *neo* insertion | TGGCGCTTACGTGCCGCTGGACCCGGATTACCCGACGGATCGACTGGCCTACATGCTGCAGGACAGCGGCGTCGAACTGCTGCCGCAAGCACTCAGGGCGCAAGG |
| oSWC4329 | Reverse PCR primer, 80 nt *pvsA*, 1 kb deletion, *neo* insertion | AGCGCCAGGGCCGATTTGATGTGCTCGGCCTGAACTTCCTGCCCGGCTTCGGCGGTGTAATAACCGATCAGTTGCGGGCCTCAGAAGAACTCGTCAAGAAGGCG |
| oSWC4330 | Forward PCR primer, 80 nt *pvsA*, 2 kb deletion, *neo* insertion | TGCTTGCCGAAGAGCTGCCGTGGCACAGCCGCGAGGCCAAGTTCGACCTGCAACTGCACAGCGAAGAGGACCGTAACGGTTGCCGCAAGCACTCAGGGCGCAAGG |
| oSWC4331 | Reverse PCR primer, 81 nt *pvsA*, 2 kb deletion, *neo* insertion | AGCGCGCCATGCCGCCGACGTTGTACGCCGGGCTGTCCGGTTCCATCTGCCAGAGGAACCACATGCGTTGTTGCGAATAGGTCAGAAGAACTCGTCAAGAAGGCG |
| oSWC4332 | Forward PCR primer, 81 nt *pvsA*, 6 kb deletion, *neo* insertion | ACCGGCCAGCCGGGGCACGGCGTGTTGATTGCCGACCCTGTCACCCTGCACGTGCTGGCTGAAAACAGCATTGGCGAAGTCTGCCGCAAGCACTCAGGGCGCAAGG |
| oSWC4333 | Reverse PCR primer, 80 nt *pvsA*, 6 kb deletion, *neo* insertion | TGACCTGCTGCTCACGAATCAGCGTGCAGATTTCTTCGGCGTCCCACTGGCCTTGCGCGCGCAATACCACGCGTGCGCCGTCAGAAGAACTCGTCAAGAAGGCG |
| oSWC4336 | Forward PCR primer, 80 nt *pvsA*, 13 kb deletion, *neo* insertion | TTTCTTGACATTCAGATCTTATCTGGC |
| oSWC4337 | Reverse PCR primer, 79 nt *pvsA*, 13 kb deletion, *neo* insertion | ATTTACACCCGGTAACGAG |
| oSWC4330 | 1301 bp insert, 80 nt *pvsA*, 2 kb deletion, *neo* insertion | TGCTTGCCGAAGAGCTGCCGTGGCACAGCCGCGAGGCCAAGTTCGACCTGCAACTGCACAGCGAAGAGGACCGTAACGGTTGCCGCAAGCACTCAGGGCGCAAGG |
| oSWC4331 | 1301 bp insert, 80 nt *pvsA*, 2 kb deletion, *neo* insertion | AGCGCGCCATGCCGCCGACGTTGTACGCCGGGCTGTCCGGTTCCATCTGCCAGAGGAACCACATGCGTTGTTGCGAATAGGTCAGAAGAACTCGTCAAGAAGGCG |
| oSWC4352 | 2263 bp insert, 80 nt *pvsA*, 2 kb deletion, *neo* insertion | TGCTTGCCGAAGAGCTGCCGTGGCACAGCCGCGAGGCCAAGTTCGACCTGCAACTGCACAGCGAAGAGGACCGTAACGGTCTGATCGTCACGGCGATTTATGCCG |
| oSWC4331 | 2263 bp insert, 80 nt *pvsA*, 2 kb deletion, *neo* insertion | AGCGCGCCATGCCGCCGACGTTGTACGCCGGGCTGTCCGGTTCCATCTGCCAGAGGAACCACATGCGTTGTTGCGAATAGGTCAGAAGAACTCGTCAAGAAGGCG |
| oSWC4353 | 3273 bp insert, 80 nt *pvsA*, 2 kb deletion, *neo* insertion | TGCTTGCCGAAGAGCTGCCGTGGCACAGCCGCGAGGCCAAGTTCGACCTGCAACTGCACAGCGAAGAGGACCGTAACGGTTGGATGCTGTAGGCATAGGCTTGG |
| oSWC4331 | 3273 bp insert, 80 nt *pvsA*, 2 kb deletion, *neo* insertion | AGCGCGCCATGCCGCCGACGTTGTACGCCGGGCTGTCCGGTTCCATCTGCCAGAGGAACCACATGCGTTGTTGCGAATAGGTCAGAAGAACTCGTCAAGAAGGCG |
| oSWC1447 | ssDNA carrier oligo | CAGTGAAAGTCGTGATGTCCAACCCTAACCCCAAGGGGAACTCGTACCCGGCAGCACGTCATGCATATGATTCGACGATTATAT |
| oSWC4347 | PCR *neo* from pK18mobsacB | CACATGCCTCAGGTGCCGCAAGCACTCAGGGCGCAAG |
| oSWC4349 | PCR *neo* from pK18mobsacB | CACATGCCTGAGGTCAGAAGAACTCGTCAAGAAGGCG |

TABLE S2. Oligonucleotides used.
